# Knowledge of PEP and PrEP among people living with HIV/aids in Brazil

**DOI:** 10.1186/s12889-020-10135-3

**Published:** 2021-01-07

**Authors:** Laelson Rochelle Milanês Sousa, Henrique Ciabotti Elias, Nilo Martinez Fernandes, Elucir Gir, Renata Karina Reis

**Affiliations:** 1 Department of General and Specialized Nursing, University of São Paulo at Ribeirão Preto College of Nursing, R. Prof. Hélio Lourenço, 3900, Vila Monte Alegre, Ribeirão Preto, SP CEP: 14040-902 Brazil; 2 Department of Nursing, Federal University of TriânguloMineiro, Uberaba, Minas Gerais Brazil; 3 Evandro Chagas Clinical Research Institute, Oswaldo Cruz Foundation, Rio de Janeiro, RJ Brazil

**Keywords:** HIV, Pre-exposure prophylaxis, Post-exposure prophylaxis, Awareness

## Abstract

**Background:**

Pre-Exposure Prophylaxis (PrEP) and Post-Exposure Prophylaxis (PEP) are key to preventing sexual transmission of HIV, whose sexual partners are at high risk of acquiring HIV.

We aimed to determine the factors associated with PrEP and PEP’s knowledge as secondary prevention among people living with HIV/AIDS.

**Method:**

Cross-sectional analytical study carried out among people living with HIV/AIDS treated at five specialized services in the city of Ribeirão Preto, São Paulo, Brazil. Data were collected from July 2016 to July 2017. Individual interviews were conducted. We used multivariable logistic regression to determine factors associated with knowing PrEP and PEP.

**Results:**

Of the 397 participants, 140 (35.26%) were heterosexual women aged 40 to 49 years (36.2%).Participants with less than 11 years of study (adjusted odds: 0.29; 95% CI: 0.13–0.60); who did not have a low viral load or did not know their viral load (adjusted odds: 0.29; 95% CI: 0.09–0.83) and those with casual partners (adjusted odds: 0.29; 95% CI: 0.09–0.83) were less likely to know about the PrEP. MSM (adjusted odds: 2.88; 95% CI: 1.59–5.3) and those who used alcohol during sexual intercourse (adjusted odds: 1.7; 95% CI: 1.0–2.8) were more likely to know about the PEP.

**Conclusions:**

The knowledge about PEP and PrEP is low in Brazil. This may undermine secondary prevention efforts. Educational interventions to raise awareness of these prevention methods are needed among people living with HIV and who have HIV-negative sexual partners.

## Background

Infection caused by the Human Immunodeficiency Virus (HIV), almost five decades after notification of the first cases, remains a worldwide public health problem, despite all the advances achieved in the treatment and expansion of prevention strategies. It is estimated that approximately 37.9 million people live with HIV worldwide by the end of 2018 [1].

In Brazil, in 2018, 43.941 new cases of HIV infection and 37.161 cases of AIDS were reported, totaling 966,058 AIDS cases detected in the country [2]. Since 1996, the country has offered antiretroviral treatment through the Unified Health System (SUS) for People Living with HIV (PLHIV) and was one of the pioneer countries among low and middle incomes. From 2013, SUS guarantees treatment for all PLHIV, regardless of the stage of the disease and the CD4 T lymphocyte count. More than 30 years after the first case, the epidemic continues to expand. New infection rates have remained persistently high over the past decade, with an estimated 48,000 new HIV infections per year, even with coverage of antiretroviral treatment (ART) in SUS [3].

The rate of AIDS detection has decreased in Brazil in recent years in the Southeast, South, and Midwest regions, while in the North and Northeast regions, they have shown an increasing trend in detection [2]. HIV infection in the Brazilian population has an estimated prevalence rate of 0.4%. However, the epidemic disproportionately affects key populations as women sex workers, men who have sex with men, and transgender women [4].

The main route of HIV transmission is sexual, among people aged 13 and over, in 2018 in all regions, both men (78.9%) and women (86.9%) [2]. In the early years of the HIV epidemic, condom use was practically the only method of preventing sexual transmission of HIV widely recommended and widespread throughout the historical path of the epidemic [5]. Condoms have advantages in low cost, easy access, and low adverse effects when they are adopted consistently and correctly. Besides, they effectively prevent other sexually transmitted infections [6] and are considered fundamental to a comprehensive approach to prevention [7].

However, this strategy does not eliminate the risk of HIV transmission. A systematic review study showed that the consistent use of condoms (for all acts of vaginal penetration) in heterosexual relationships results in an 80% reduction in the incidence of HIV [8] and 70% among male homosexuals (for anal sex) [9]. Studies have also shown inconsistent condom use among partners living with HIV and relating to HIV-negative people or with unknown HIV status [6, 10].

A study conducted in Brazil identified that PLHIV undergoing clinical-outpatient follow-up who had less education, multiple sexual partners, using alcohol or other drugs, do not receive advice from a healthcare professional. They have no knowledge of treatment as prevention. Not knowing that undetectable viral load reduces the risk of human immunodeficiency virus transmission was associated with inconsistent condom use [6], which reflects the difficulty in accessing health information to assimilate the orientations received and to change health behavior.

Also, aspects related to social gender norms determine a low power of sexual negotiation for Brazilian women making them more likely to have unprotected sex. Despite the advancement of feminist movements, women’s role in sexuality and their responsibility for reproductive issues hinders dialogue with their partners and increases vulnerability [11].

Gender-based violence reduces engagement for women living with HIV at multiple care continuum levels [12]. It might be a particularly salient issue for the Latin American region, where high rates of intimate partner violence, sexual assaults, and femicide have been documented [13, 14].

Therefore, important advances in the field of HIV prevention have provided paradigm shifts with the implementation of combined biomedical, behavioral, and structural interventions [15]. Combined prevention is a broader concept that combines different prevention methods that can interfere with the sexual transmission of the virus, with the use of antiretrovirals, including Pre-Exposure Prophylaxis (PrEP) and Post-Exposure Prophylaxis (PEP), termed as biomedical interventions. Such strategies have been considered to be effective in reducing the risk of HIV transmission and are part of combined prevention [16].

As a result of advances in HAART, post-sexual exposure to HIV prophylaxis (PEP) was implemented in 2012, is a method used in situations where sexual exposure to HIV occurs, especially when the sexual act was performed in the absence of condoms, or in times of condom failures, such as breakage or problems with structural characteristics due to inappropriate use. Their use can reduce the risk of acquiring the infection through a therapeutic regimen with antiretrovirals [16].

PrEP was implemented in Brazil by the public health system on December 1, 2017 [13] and is an important advance in prevention and is used before exposure to the virus, recommended for homosexuals, men who have sex with men (MSM), transgender, sex workers, people who use drugs, those who are incarcerated, serodiscordant partnerships, taking into account also the repetitions of anal and/or vaginal sexual practices with penetration without the use of condoms, frequency of sexual relations with casual partners, quantity and diversity of sexual partnerships, contexts of transactional sex (for money, valuables, housing, drugs, among others), history of sexually transmitted infections and repeated search for post-exposure prophylaxis (PEP) [17–19].

Although these two biomedical interventions based on drug treatment are considered essential for prevention, there are gaps in the knowledge of both HIV-negative/unknown people and among PLHIV. When revisiting studies that investigated the awareness of PrEP and PEP, it was found that the levels were below expectations. Even with the release of the use of PrEP in the United States [5]. Similar results were observed among Nigerian university students [14]. In Canada, MSM demonstrated incipient knowledge about PrEP, including those who were HIV-negative and HIV-positive [14].

To achieve relevant results in HIV prevention, through the use of such strategies, it is necessary to expand awareness and use education actions, focusing on people most exposed to the virus [20], such as sexual partners, whether fixed or casual, of PLHIV. Besides, expanding education actions for the general population may contribute to adherence to HIV prevention methods.

In general, knowledge about PrEP and PEP is surprisingly low, both in developed countries [21–23] and in developing countries, as in Brazil [23]. However, studies conducted in three countries in Latin America and the United States of America show that interest in using PrEP is high among key populations [5, 24].

Antiretroviral drugs, used as antiretroviral therapy (ART) and PrEP, are powerful HIV prevention tools for HIV serodiscordant couples. Although the HIV prevention effectiveness of ART and PrEP is proven, the prevention benefits are only realized when adherence is high [25]. Also, the residual risk of HIV transmission persists during the first 6 months of ART, with incomplete viral suppression in blood and genital compartments. For HIV-serodiscordant couples in which the infected partner is starting ART, other prevention options are needed, such as pre-exposure prophylaxis, until viral suppression is achieved [26].

In this context, current World Health Organization [27] guidelines recommend ART for all HIV positive adults diagnosed with HIV and PrEP as part of the HIV prevention combination for people at substantial risk of HIV, including HIV negative partners of couples. Due to the scarcity of studies that address the theme in Brazil, this study aimed to analyze the factors associated with the knowledge of PEP and PrEP as secondary prevention among people living with HIV/AIDS to improve policies for the implementation and distribution of strategies in a broader prevention plan among people at risk of infection.

## Methods

This is a cross-sectional and analytical study conducted with people living with HIV undergoing clinical-outpatient follow-up at five health services in a city in the interior of the state of São Paulo, Brazil. The health services in which the study was conducted are specialized in serving people living with HIV.

Participants needed to meet the following criteria to be included in the study: being aware of the diagnosis of HIV infection; being 18 years of age or older; being under clinical-outpatient monitoring at selected health services; have an active sex life and a seronegative or unknown serological status partner for HIV in the last 6 months. Also, they were excluded if they were in confinement, regardless of the type of institution.

Data were collected from July 2016 to July 2017. Individual interviews were conducted, in a private environment, by researchers trained for this function. The participants’ approach occurred at the time of their visits to the health services, and the interviews were scheduled according to the time chosen by the participant, before or after the medical consultation, if they agreed to participate. The interviews’ average duration was 30 min and only started after the participant gave their free and informed consent in writing.

The sample size was estimated from the approximate number of people with active records in the municipality’s specialized services where the study was conducted. Another parameter was the estimate of sexually active people living with HIV after diagnosis (62%). Therefore, the sample was set at 286. The calculation was performed with the aid of software R version 3.4.1. However, more people were included in the study to avoid potential losses from invalid or incomplete questionnaires.

The independent variables of the study were: sexual orientation, age, skin color, education (in years of study), work situation, length of HIV diagnosis, type of partner, partner’s serology, use of alcohol during sexual intercourse, use of other drugs during sexual intercourse and condom use. The dependent variables were: having knowledge about PrEP (yes, no) and having knowledge about PEP (yes, no).

The data were tabulated in Microsoft Excel 2010, and after double typing, the databank was exported to the Statistical Package for the Social Sciences (SPSS), version 22.0. Descriptive statistics were used to characterize the sample. Association tests were performed (Chi-square and Fisher’s Exact), considering a statistical significance level at *p* < 0.05.

Logistic regression analysis was used to assess the influence of independent variables on dependent variables, to have knowledge about PrEP and PEP. The automatic variable selection procedure called “Stepwise” was used for the selection of independent variables, using the Akaike Information criterion [28]. For the analysis, the significance level of 5% (α = 0.05) was used. The programs used in the analyzes were SPSS (IBM Corp. Released, 2013) version 22 and R (R Core Team, 2018) version 3.5.3.

The Research Ethics Committee approved the study of the Ribeirão Preto School of Nursing under protocol 084/2016 and CAAE: 52012515.0.00000.5393. All participants signed informed consent forms. The researchers guaranteed the participants anonymity.

## Results

This study included 397 people living with HIV. It is noteworthy that 140 (35.3%) were heterosexual women, 136 (34.3%) were men who have sex with men (MSM). 147 (37%) aged 35–44 years and 214 (53.9%) with less than 11 years of study. Notably, 254 (64%) used condoms consistently.

As for knowledge about PrEP, being MSM was a factor associated with having knowledge (*p* = 0.000). Having less than 11 years of study was associated with not having knowledge about PrEP (p = 0.000), according to Table 1.

**Table 1:** Sociodemographic and behavioral variables and knowledge aboutPrEP. Ribeirão Preto, SP, 2020

Characteristics	Knowledge of PrEP			
Sexual orientation	Total	Yes	No	***P*** Value
Heterosexual woman	140 (35.3%)	11 (18.6%)	129 (38.5%)	0.001
Heterosexual man	121 (30.5%)	13 (22%)	106 (31.6%)	
MSM	136 (34.3%)	35 (59.3%)	100 (29.9%)	
**Age**				
18–24	30 (7.6%)	5 (8.5%)	25 (7.5%)	0.417
25–34	87 (21.9%)	18 (30.5%)	68 (20.3%)	
35–44	147 (37%)	21 (35.6%)	124 (37%)	
45+	133 (33.5%)	15 (25.4%)	118 (35.2%)	
**Education**				
< 11 years	214 (53.9%)	13 (22%)	199 (59.4%)	0.001
> 11 years	183 (46.1%)	46 (78%)	136 (40.6%)	
**Race**				
White	210 (53.3%)	32 (54.2%)	176 (52.9%)	0.407
Not white	184 (46.7%)	27 (45.8%)	157 (47.1%)	
**Employment status**				
Employed	259 (65.2%)	43 (72.9%)	213 (63.6%)	0.621
Unemployed	84 (21.2%)	11 (18.6%)	73 (21.8%)	
Others	49 (12.3%)	4 (6.8%)	45 (13.4%)	
Unable to work	5 (1.3%)	1 (1.7%)	4 (1.2%)	
**Time since HIV diagnosis (years)**				
< 2 to 2–4.9	154 (38.8%)	26 (44.1%)	127 (37.9%)	0.657
≥ 5	243 (61.2%)	33 (55.9%)	208 (62.1%)	
**Type of partner**				
Fixed	255 (64.2%)	41 (69.5%)	213 (63.6%)	0.579
Casual	125 (31.5%)	15 (25.4%)	108 (32.2%)	
Fixed/Casual	17 (4.3%)	3 (5.1%)	14 (4.2%)	
**Serology of partner**				
HIV-positive	111 (28%)	10 (16.9%)	101 (30.1%)	0.064
HIV-negative/unknown	286 (72%)	49 (83.1%)	234 (69.9%)	
**Use of alcohol during sex**				
Yes	169 (42.6%)	24 (40.7%)	142 (42.4%)	0.126
No	228 (57.4%)	35 (59.3%)	193 (57.6%)	
**Use of drugs during sex**				
Yes	76 (19.1%)	8 (13.6%)	67 (20%)	0.419
No	321 (80.9%)	51 (86.4%)	268 (80%)	
**Use of condom**				
Consistent	254 (64%)	46 (78%)	208 (62.1%)	0.004
Inconsistent	143 (36%)	13 (22%)	127 (37.9%)	

Source: The authors

It is noteworthy that being an MSM, having more than 11 years of study, and being employed were associated with knowledge about PrEP (*p* = 0.001), according to Table 2.

**Table 2:** Sociodemographic and behavioral variables and knowledge about PEP. Ribeirão Preto, RP, 2020

Characteristics	Knowledge of PEP			
	Total	Yes	No	***P*** Value
**Sexual orientation**				
Heterosexual woman	140 (35.3%)	25 (20.5%)	115 (41.8%)	0.001
Heterosexual man	121 (30.5%)	29 (23.8%)	92 (33.5%)	
MSM	136 (34.3%)	68 (55.7%)	68 (55.7%)	
**Age**				
18–24	30 (7.6%)	11 (9%)	19 (6.9%)	0.066
25–34	87 (21.9%)	36 (29.5%)	51 (18.5%)	
35–44	147 (37%)	39 (32%)	108 (39.3%)	
45+	133 (33.5%)	36 (29.5%)	97 (35.3%)	
**Education**				
< 11 years	214 (53,9%)	43 (35.2%)	171 (62.2%)	0.001
> 11 years	183 (46,1%)	79 (64.8%)	104 (37.8%)	
**Color**				
White	210 (53.3%)	69 (57%)	141 (51.6%)	0.324
Not white	184 (46.7%)	52 (43%)	132 (48.4%)	
**Employment status**				
Employed	259 (65.2%)	94 (77%)	165 (60%)	0.007
Unemployed	84 (21.2%)	18 (14.8%)	66 (24%)	
Others	49 (12.3%)	8 (6.6%)	41 (14.9%)	
Unable to work (incapacity/disability	5 (1.3%)	2 (1.6%)	3 (1.1%)	
**Time since HIV diagnosis (years)**				
< 2 to 4.9	154 (38.8%)	47 (38.5%)	107 (38.9%)	0.942
≥ 5	243 (61.2%)	75 (61.5%)	168 (61.1%)	
**Type of partner**				
Fixed	255 (64.2%)	72 (59%)	183 (66.5%)	0.353
Casual	125 (31.5%)	44 (36.1%)	81 (29.5%)	
Fixed and casual	17 (4.3%)	6 (4.9%)	11 (4%)	
**Serology of partner**				
HIV-positive	84 (28%)	18 (20.5%)	66 (31.1%)	0.061
HIV-negative/unknown	216 (72%)	70 (79.5%)	146 (68.9%)	
**Use of alcohol during sex**				
Yes	169 (42.6%)	62 (50.8%)	107 (38.9%)	0.027
No	228 (57.4%)	60 (49.2%)	168 (61.1%)	
**Use of drugs during sex**				
Yes	76 (19.1%)	29 (23.8%)	47 (17.1%)	0.119
No	321 (80.9%)	93 (76.2%)	228 (82.9%)	
**Use of condom**				
Consistent	254 (64%)	77 (63.1%)	177 (64.4%)	0.811
Inconsistent	143 (36%)	45 (36.9%)	98 (35.6%)	

Source: The authors

As for the factors associated with PrEP, participants with less than 11 years of study were (adjusted odds: 0.29; 95% CI: 0.13–0.60) less likely to have knowledge about PrEP compared to participants with more than 11 years of study. Those who did not have a low viral load or did not know their viral load (adjusted odds: 0.26; 95% CI: 0.09–0.83) were less likely to have knowledge about PrEP compared to those with low viral load. Likewise, those with casual partners had (adjusted odds: 0.29; 95% CI: 0.09–0.83) less chance (or a 71% lower chance (1–0.29) of having knowledge about PrEP compared with those who were in a fixed relationship, as shown in Table 3.

**Table 3:** Adjusted model of factors associated with knowledge of PrEP. Ribeirão Preto, SP, 2020

Variable	Crude odds [95% CI]	***P*** Value	Adjusted Odds [95% CI]	***P***-Value
**Education (years)**				
< 11	0.19 (0.1–0.37)	**0.001**	0.29 (0.13–0.60)	0.001
**Sexual orientation**				
Heterosexual Women	0.69 (0.3–1.6)	0.386	0.77 (0.30–1.9)	0.591
MSM	2.88 (1.44–5.77)	**0.003**	2.08 (0.97–4.6)	0.065
**Low viral load**				
No/Unknown	0.3 (0.16–0.55)	**0.001**	0.26 (0.13–0.50)	0.001
**Type of partner**				
Casual	0.71 (0.38–1.34)	0.293	0.29 (0.09–0.83)	0.025
Fixed/ Casual	1.1 (0.3–3.99)	0.888	0.25 (0.04–1.19)	0.098
**Status of partner**				
Negative/Unknown	2.05 (1,4.21)	0.050	2.12 (0.95–5.0)	0.074
**Number of sexual partners**				
Multiple partners	1.48 (0.83–2.65)	0.182	2.3 (0.93–6.22)	0.070
**Conversations with partner**				
No	0.41 0.23–0.76)	**0.004**	0.54 (0.25–1.14)	0.118

Source: The authors

As for the factors associated with PEP, MSM participants were 2.88 (Adjusted Odds: 2.8788; 95% CI: 1.5912–5.3092) times more likely to have knowledge about PEP than heterosexual male participants. Participants who used alcohol during intercourse were 1.73 (adjusted odds: 1.7305; 95% CI: 1.0656–2.8248) times more likely to have knowledge about PEP than those who did not use alcohol during intercourse.

Participants with less than 11 years of education (adjusted Odds: 0.4331; 95% CI: 0.2564–0.7271) were less likely to have knowledge about PEP compared to participants with more than 11 years of education. Participants who did not have a low viral load or did not know about their viral load (adjusted odds: 0.4054; 95% CI: 0.2493–0.6511) were less likely to have knowledge about PEP than those with low viral load, as shown in Table 4.

**Table 4:** Adjusted model of the factors associated with the knowledge of PEP. Ribeirão Preto, RP, 2020

Variable	Crude odds [95% IC]	***P***-Value	Adjusted odds [95% IC]	***P***-value
**Sexual orientation**				
Heterosexual woman	0.73 (0.4–1.35)	0.327	0.90 (0.47–1.7)	0.764
MSM	3.32 (1.92–5.74)	0.001	2.8 (1.5–5.3)	**0.006**
**Education**				
< 11	0.33 (0.2–0.51)	0.001	0.43 (0.25–0.72)	**0.001**
**Time since HIV diagnosis**				
Five years or more	0.99 (0.6–1.5)	0.977	1.4 (0.88–2.4)	0.139
**Low VL**				
No/Unknown	0.42 (0.2–0.65)	0.001	0.40 (0.24–0.65)	**0.001**
**Use of alcohol during sex**				
Yes	1.58 (1.02–2.43)	0.041	1.7 (1.0–2.8)	**0.027**

Source: The authors

## Discussion

Participants in this study had low knowledge of PrEP. People with less than 11 years of study, who did not have a low viral load or did not know about viral load rates, who had fixed partners, were less likely to have knowledge of PrEP. As for the knowledge about PEP, it was identified that MSM and individuals who reported using alcohol during sexual intercourse were more likely to have knowledge about PEP.

International evidence corroborates these findings and indicates that PrEP and PEP awareness is low, even among key populations that are considered a priority for infection control, the percentage of participants who had knowledge about either of the two methods ranges from 18.9 to 47.2% [14, 29]. Besides, a study with MSM identified low awareness of the two methods but a high level of interest in using them [5].

In general, priority populations do not have satisfactory knowledge about sexually transmitted infections and prevention methods. For example, in 12 Brazilian cities, 4176 MSM were recruited to evaluate the knowledge of these individuals about HIV/AIDS. The proportion who had a high level of knowledge was 23.7%, and those with 12 or more years of study were linked [30]. MSM are a key population and, therefore, have access rights to PrEP, and as far as PEP is concerned, it is recommended in specific cases [31]. However, these populations must have knowledge about the infection and its main forms of prevention so that they have access to the methods and can discuss them with their sexual partners.

Some facts have been reported in the literature as barriers to adherence to PrEP. These issues were noted in 2004 when the PrEP movement started in the United States. The volunteers were the target of prejudice with derogatory expressions aimed mainly at gay men. This association between promiscuity and homosexuality also happened in Brazil with headlines from magazines with high circulation. This view can provide individuals with distance from the possibilities of coping with new infections and the quality of life for those who live with HIV [31]. In this sense, the need is emphasized for populations at high risk of infections to have access to quality information about the infection and its prevention and control methods.

A higher level of education was associated with having knowledge about PEP among PLHIV/AIDS residing in Italy. Among MSM, a higher level of education remained an associated factor [32]. Data from a study conducted in the United States showed that attending a college or a higher education course was associated with awareness of PrEP [33]. Such a finding provokes interest in the construction of health education strategies that can reach people with a lower level of education through the provision of information that meets their needs in terms of understanding and access to information, especially with instructions that present the method to those with no knowledge [30].

PLHIV/AIDS and their sexual partners are central to controlling the epidemic [10, 34]. Therefore, providing support to PLHIV strengthens the health professional’s bond and can expand their participation in health promotion activities. Community support was found to be relevant for a group of MSM and women. Those who received support were more likely to be aware of PrEP [35]. Building paths to enrich the knowledge of PLHIV about the infection seems to be a challenge that deserves the engagement of governmental and non-governmental entities, health professionals, and civil society to overcome barriers that can hinder access to all forms of prevention available.

The use of alcohol during sexual intercourse was shown to be positive given PEP’s knowledge. It is worth mentioning that the use of alcohol in sexual relations makes the individual more vulnerable, including sexual practices without protection [36]. Another important contribution to this discussion is the belief in the toxicity of antiretrovirals with alcohol. That is, to believe that when consuming alcoholic beverages, there may be a decrease in the effect of the ingested medicine. A study on beliefs related to PrEP reported that 75% of participants had beliefs about interactive toxicity between alcohol and antiretrovirals [37].

In this sense, researchers highlighted that the forms of disclosure of PrEP could include messages that generate stigma against its users. A comprehensive view of health should guide strategies to include people without segregating them into pre-existing groups tied to old stigmas [38]. Other studies have corroborated the idea that adherence to PrEP is hindered by stigma, even though its central function is to prevent sexual transmission of HIV [39].

This study has limitations. The fact that this research did not include the affective/sexual partnerships of PLHIV who have negative/unknown serology reduces the understanding of the knowledge of the two methods addressed and centralizes the discussion of the approach to prevention from the perspective of PLHIV/AIDS. Therefore, future research should include sexual partnerships and investigate the complexity involved in negotiating the use of different methods to prevent sexual transmission of the virus.

## Conclusions

Knowledge about PrEP and PEP is low among PLHIV in Brazil. People with less than 11 years of study, who did not have a low viral load or did not know their viral load, with casual partners, were less likely to have knowledge about PrEP. Knowledge about PEP was more satisfactory. MSM and participants who used alcohol during sexual intercourse were more likely to have knowledge about PEP. Therefore, providing health education at appropriate times for PLHIV reinforces their knowledge of these methods and may arouse interest in including different prevention strategies in their daily relationships with affective-sexual relationships with negative/unknown status partners.

## Data Availability

The data sets used and/or analyzed during the present study are available with the corresponding author upon reasonable request.

## Abbreviations

HIV: Human Immunodeficiency Virus (HIV)
AIDS: Human Immunodeficiency Syndrome
PLHIV: people living with HIV
PrEP: Pre-Exposure Prophylaxis
PEP: Post-Exposure Prophylaxis
MSM: men who have sex with men
SPSS: Statistical Package for the Social Sciences
